# Universal video steganalysis in HEVC intra frame based on the convolutional residual network

**DOI:** 10.3389/frai.2026.1877600

**Published:** 2026-09-15

**Authors:** Mingyuan Cao, Lihua Tian, Chen Li

**Affiliations:** School of Software Engineering, Xi'an Jiaotong University, Xi'an, China

**Keywords:** block partitioning structure, convolutional neural network, intra prediction mode, transform block decision, universal video steganalysis

## Abstract

High Efficiency Video Coding (HEVC) offers a variety of suitable carriers for video steganography, contributing to the rapid advancement of the field. However, most of the existing steganalysis algorithms can only detect a single type of video steganography and are unable to detect other types of video steganography. To detect different video steganography methods in multiple domains of intra frame, a universal video steganalysis algorithm based on convolutional residual networks is proposed in this paper. Firstly, we introduce and analyze three types of video steganography in intra frame: video steganography based on intra partition structures, transform unit (TU) partition modes and intra-prediction modes (IPM). It reveals that these types of algorithms have minimal impact on the spatial domain of the video. However, they may disrupting the optimality of the local structure or the intra-prediction modes in specific regions of the video. Building on this observation, we introduce the concept of the IPM map, which not only effectively captures the IPM information of the video frame but also partially reflects the partition structure information of the video. Then, the IPM maps from both the original and recompressed video frame can be extracted. The difference between these two maps serves as the input to the convolutional residual network. Finally, a convolutional residual network, called IFSN (Intra Frame Steganalysis Network), comprising preprocessing, feature extraction, feature representation and binary classifier, is used for training and detecting the video frames. Experimental results demonstrate that the proposed convolutional residual network effectively detects different video steganography algorithms in multiple domains of intra frame.

## 1 Introduction

Steganography is a key technique in the field of information hiding, aimed at embedding confidential messages imperceptibly within digital media to enable covert communication. In contrast, steganalysis focuses on detecting the presence of such concealed information. Common media employed for steganography include text, audio, images, and videos (Karampidis et al., 2018; Wang et al., 2020; Ekodeck and Ndoundam, 2016; Tang et al., 2019; Abdollahi et al., 2023; Yousefi Valandar et al., 2022). With the rapid growth of video-based platforms, video content has emerged as a predominant form of digital communication. This shift has heightened the importance of safeguarding video data, prompting increased research efforts to develop both video steganography and steganalysis methods as effective tools for multimedia security.

As the successor to the widely utilized H.264/AVC standard, High Efficiency Video Coding (HEVC) delivers improved video quality and significantly better compression efficiency. These advantages have led to its broad deployment in areas such as high-definition digital television, online video streaming, and video conferencing in HD. In addition to its core use cases, HEVC has also played a pivotal role in advancing video compression techniques, thereby providing fertile ground for the development of video steganographic methods. However, this rapid advancement has concurrently underscored the urgent demand for more sophisticated video steganalysis techniques.

Throughout the HEVC encoding pipeline, multiple syntax elements are commonly exploited for the purpose of steganographic embedding. These include motion vectors, inter prediction strategies, intra-frame prediction modes, transform coefficients such as DCT and DST, entropy coding schemes, and the hierarchical block partitioning structure.

In the field of quantized transform coefficients-based steganography, Ma et al. (2010) developed an efficient approach for handling the quantized discrete cosine transforms (QDCTs) in 4 × 4 luminance blocks within the H.264 standard. Subsequently, Lin et al. (2013) proposed a technique that achieved greater embedding capacity compared to Ma's method. Building on this, Chen et al. (2018) designed an adaptive embedding framework to further improve capacity, and Nguyen et al. (2019) extended this advancement by significantly increasing embedding performance. Moreover, extensive research has also been conducted on data hiding techniques for the HEVC standard (Tew et al., 2016; Chang et al., 2014; Liu et al., 2019). Among these, Chang et al. (2014) presented the pioneering DCT-based embedding algorithm for HEVC, while Liu et al. (2019) proposed an innovative method employing 4 × 4 luminance discrete sine transform blocks to mitigate intra-frame distortion drift.

In the domain of Intra Prediction Mode-based steganography, initial investigations (Hu et al., 2007; Yang et al., 2011; Bouchama et al., 2012) primarily targeted the H.264/AVC standard. These works concealed data by classifying the nine directional prediction modes of H.264/AVC to encode secret message. Hu et al. (2007) introduced a steganographic technique utilizing intra prediction modes within H.264/AVC. Subsequently, Yang et al. (2011) refined Hu's approach by incorporating matrix coding to enhance efficiency. Bouchama et al. (2012), on the other hand, achieved superior video quality while preserving a high embedding capacity. In the context of HEVC, Jian et al. (2015) directed their efforts toward optimizing the embedding distortion across 35 IPM directions. They enhanced performance by separately incorporating angular and non-angular modes. Wang et al. (2018) devised unique STC distortion functions tailored for visual quality and compression efficiency, resulting in improved performance. Xing et al. (2024) embedded the secret messages by modifying both IPM and TU partition modes with higher capacity. Dong et al. (2023b) proposed an adaptive steganography in multi-channel.

Block partitioning-based steganography has gained attention due to its high visual quality and substantial embedding capacity, establishing it as a distinct form of steganography. Xiao-Yuan et al. (2012) proposed leveraging macroblock MB partitions in H.264/AVC for message embedding. Tew and Wong (2014) introduced a method that manipulates the block partitioning of CTUs, embedding secret information by modifying the structure of 8 × 8 CUs. In Dong's approach Dong et al. (2023a), secret messages are embedded across multiple types of CUs using a structure merging strategy.

In contrast to the diversity of video steganographic approaches, research on video steganalysis remains relatively underdeveloped. A number of steganalysis techniques have been proposed to identify DCT coefficient-based embedding, with most emphasizing the spatial-domain correlations among pixels. Shi et al. (2021) employed image steganalysis-derived features to identify alterations in statistical distributions. Building upon this, Wang P. et al. (2017) adopted video DCT residual features–adapted from traditional DCT residuals–to distinguish stego videos from their cover counterparts. Furthermore, Wang Y. et al. (2017) introduced a detection framework grounded in the centralized error distribution model to effectively identify DCT coefficient-based steganography.

In steganalysis of inter prediction mode-based steganography, most studies have focused on constructing global statistical feature representations. Li et al. (2019) proposed a 25-dimensional statistical feature set that detects steganographic content by analyzing the distribution patterns of PU partition modes. Building on this, Huang et al. (2020) introduced a 24-dimensional joint feature set incorporating calibration techniques to improve the detection of PU-based steganography. More recently, Dai et al. (2023) developed a multi-scale convolutional residual network to effectively identify hidden information embedded within PUs.

Regarding steganalysis approaches for intra prediction modes, Liu and Li (2020) designed a convolutional neural network-based framework to detect IPM-based steganography. Expanding upon the calibration principle, Zhao et al. (2016) presented an IPM shift-based detection method capable of identifying IPM steganography in both AVC and HEVC standards.

So far, no existing steganalysis techniques have been specifically designed to detect steganography based on intra block partitioning structures. This form of steganography does not modify critical encoding elements such as intra prediction modes, motion vectors, PU partitioning patterns, or quantization coefficients. In contrast, most conventional video steganalysis methods rely on examining these features to uncover hidden information. Consequently, steganographic schemes that manipulate block partitioning structures–while leaving standard encoding features intact–present a considerable challenge for detection using current steganalysis approaches.

In addition, most of the existing steganalysis algorithms can only detect a single type of video steganography and are unable to detect other types of video steganography. To detect different video steganography methods in multiple domains of intra frame, a universal video steganalysis algorithm based on convolutional residual networks is proposed in this paper. Firstly, we introduce and analyze three types of video steganography in intra frame: video steganography based on intra partition structures, TU partition modes and intra-prediction modes (IPM). It reveals that these types of algorithms have minimal impact on the spatial domain of the video. However, they may disrupting the optimality of the local structure or the intra-prediction modes in specific regions of the video. Building on this observation, we introduce the concept of the IPM map, which not only effectively captures the IPM information of the video frame but also partially reflects the partition structure information of the video.Then, the IPM maps from both the original and recompressed video frame can be extracted. The difference between these two maps serves as the input to the convolutional residual network. Finally, a convolutional residual network, called IFSN (Intra Frame Steganalysis Network), comprising preprocessing, feature extraction, feature representation and binary classifier, is used for training and detecting the video frames. Experimental results demonstrate that the proposed convolutional residual network effectively detects different video steganography algorithms in multiple domains of intra frame.

The key contributions of this paper are summarized as follows:

1. Most of the existing video steganalysis algorithms can only detect one single type of video steganography and are unable to detect other types of video steganography. To detect different video steganography methods in multiple domains of intra frame, a universal video steganalysis algorithm based on convolutional residual networks is proposed in this paper.
2. After analyzing the impact of three different types of video steganography. It is observe that, compared to cover video, the partitioning structure and IPM of stego video exhibit changes in the trends of intra partition structure and intra prediction mode after calibration. Therefore, IPM map is proposed for steganalysis. IPM map not only effectively captures the IPM information of the video frame but also partially reflects the partition structure information of the video. This allows the convolutional residual network to effectively detect different types of video steganography methods.
3. The difference between the IPM maps of the original video and its calibrated video is used as input to the convolutional residual network. This network consists of three components: feature extraction, feature representation, and binary classification. Experimental results demonstrate that, compared with existing video steganalysis methods, the proposed convolutional residual network can effectively detect intra-video steganography in multiple domains.

## 2 Preliminaries and analysis

In this section, we first introduce the intra partitioning structure in HEVC. then we analyze the effect of different types of steganography on the cover video.

### 2.1 Partitioning structure in HEVC

HEVC integrates several advanced techniques that enable it to reduce bitrate by nearly 50% without compromising video quality. The standard introduces novel block coding structures, including CTUs, CUs, PUs, and TUs, providing increased flexibility in video compression. These coding elements range in size from 8 × 8 to 64 × 64, offering a wider selection of encoding options compared to H.264's more limited unit sizes. As a result, H.265 can employ larger coding units to efficiently compress smooth areas of the video, thereby lowering bitrate, while smaller units are used for complex or highly textured regions, preserving visual fidelity.

Within the H.265 video compression framework, each frame is divided into multiple non-overlapping CTUs, each typically measuring 64 × 64 pixels. A CTU may operate as a single CU or be recursively split into smaller CUs—such as 32 × 32, 16 × 16, or 8 × 8—according to the procedure outlined in Algorithm 1. The partitioning mechanism can be conceptually separated into two distinct sub-processes. The first, referred to as the Downward Recursive Process (DRP), corresponds to steps 1 through 8 in Algorithm 1. The second, termed the Upward Recursive Process (URP), encompasses steps 9 to 15 as described in Dong et al. (2023a).

Algorithm 1 Intra Blocking Partitioning Process 
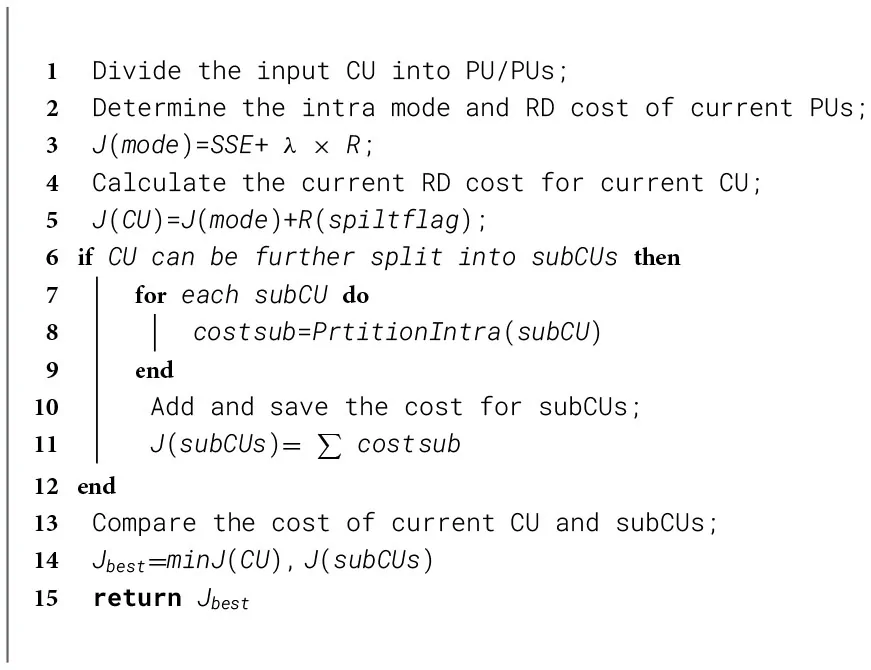

In the context of the DRP, a single CTU is initially treated as a whole CU during the intra prediction process. In the HEVC standard, the Rate Distortion Optimization (RDO) method is employed to balance compression efficiency with distortion. During the intra coding procedure, the Sum of Absolute Transformed Differences (SATD) is applied to evaluate 35 potential intra prediction directions, as SATD serves as a reasonable indicator of the bitstream size. The SATD value is determined as follows:


$$\begin{array}{l} SATD=\underset{x\in Res}{\sum }\underset{y\in Res}{\sum }|\frac{H\left(Res\left(x,y\right)\right)}{2}| \end{array}$$ (1)

where *Res*(*x, y*) denotes the residual signal corresponding to the current block, while *H*(·) represents the Hadamard transform operation. After identifying several intra prediction modes with the lowest SATDs, these candidate directions, together with the Most Probable Modes (MPMs), are subjected to the RDO process to compute the Rate-Distortion cost, expressed as *J*(*mode*).


$$\begin{array}{l} J\left(mode\right)=SSE+\lambda \times R \end{array}$$ (2)

where *SSE* represents Sum of Squared Error operation, with the Lagrangian multiplier being represented by λ, and “mode” refers to the prediction direction. The term *R* corresponds to the actual bitstream size for the given block. The RD costs are calculated using Equation 1. By comparing the RD costs associated with all possible modes, the mode yielding the minimum RD cost is selected. Unlike earlier video encoding standards, the RDO technique is not only applied to the intra prediction mode selection but is also extended to CU block partitioning, owing to its recursive nature. The RD cost for a given CU, denoted as *J*(*CU*), is defined as:


$$\begin{array}{l} J\left(CU\right)=J\left(mode\right)+R\left(spiltflag\right) \end{array}$$ (3)

where, *R*(*splitflag*) represents the number of bits required to encode the split flag. During the DRP, the RD cost of each CU at different depth levels within a CTU is computed.

In the subsequent URP, the encoder determines the final block partitioning configuration based on the RD costs of all candidate CUs. The combination of CUs yielding the minimum overall RD cost is selected and retained as the final partition structure.

As illustrated in Figure 1, the CTU partitioning process begins with a CTU at depth zero, which is initially divided into four CUs of 32 × 32 at depth one. Among these, the seventh and eighth CUs remain unsplit, while the remaining two undergo further subdivision into four smaller CUs of 16 × 16 at depth two. Subsequently, two of the eight 16 × 16 CUs are again partitioned into 8 × 8 CUs at depth three, completing the hierarchical division process.

**Figure 1:**
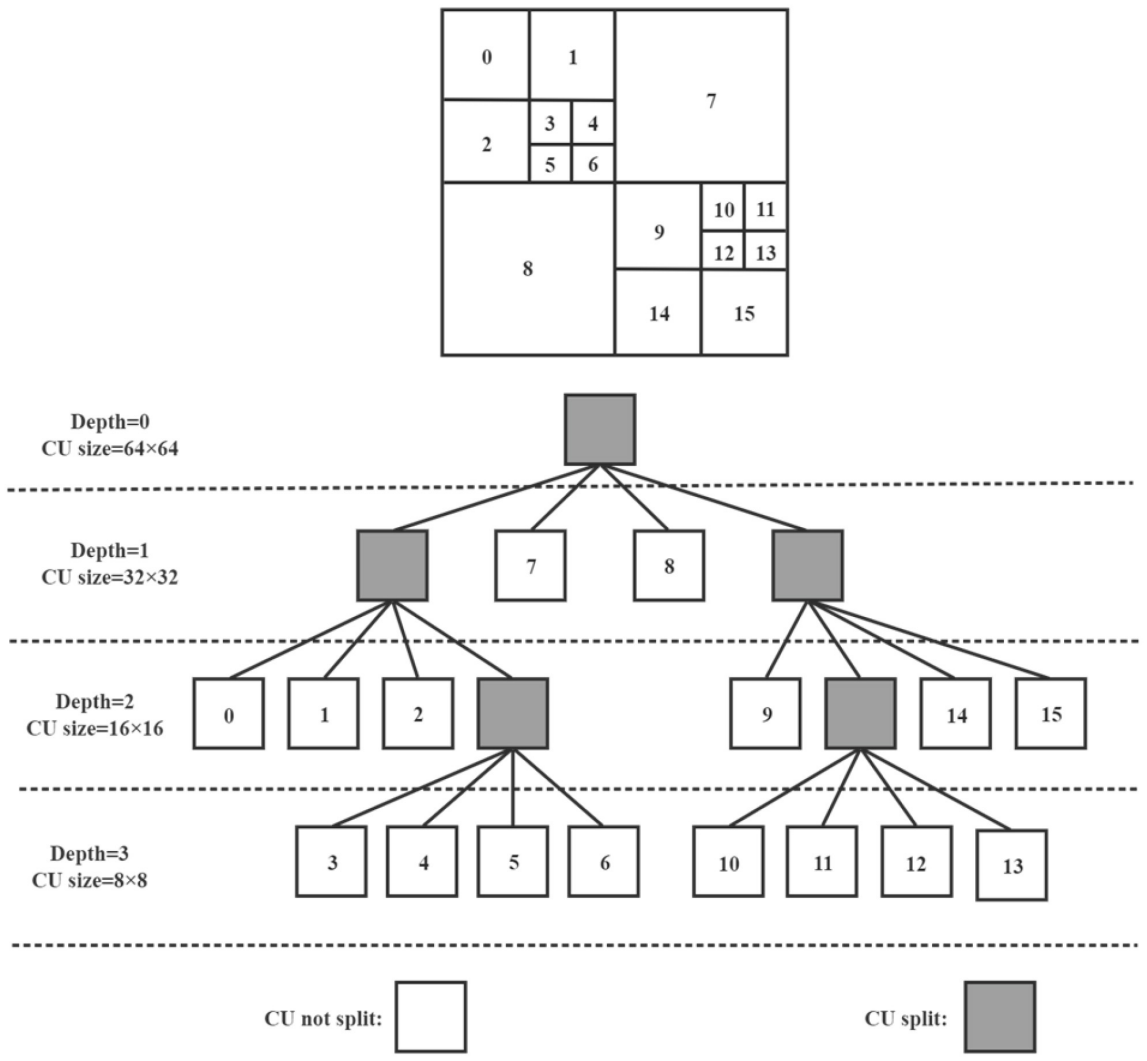
An example of CU partition.

Each CU can be further divided into PUs and TUs). As shown in Figure 2, a CU supports eight distinct PU partition configurations. While all eight partition modes are applicable to inter prediction, only two partition modes are utilized for intra prediction: N × N and 2N × 2N modes. The optimal partitioning structure for a CU is determined through the RDO process, which seeks to balance encoding bit consumption with the resulting video quality degradation.

**Figure 2:**
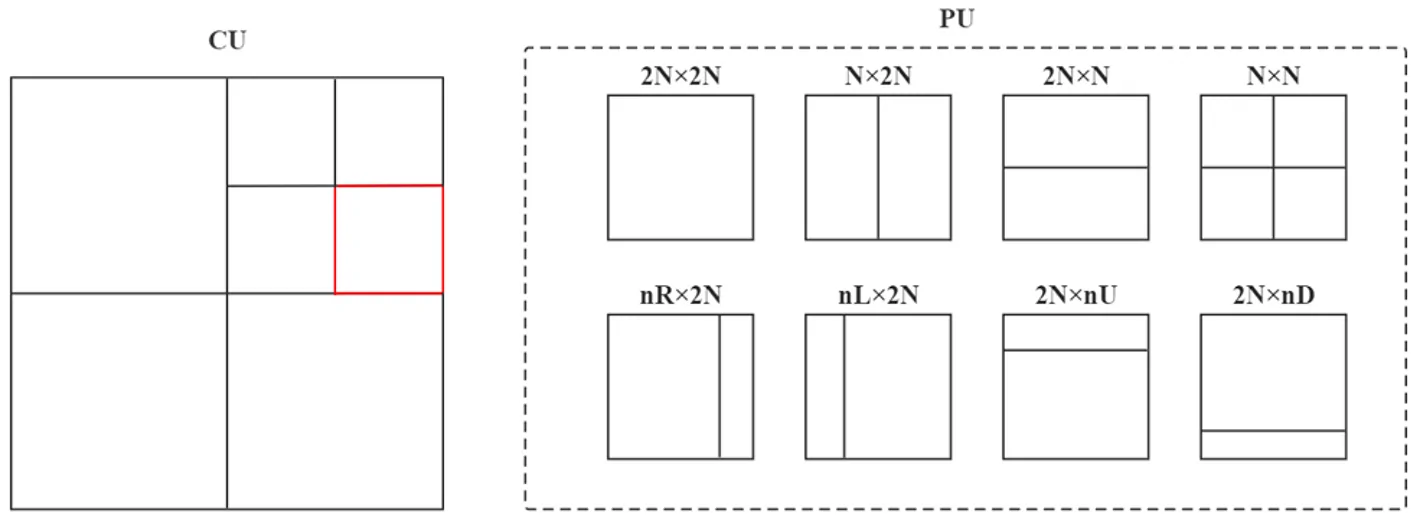
Partition modes of PU.

TUs act as the basic elements for residual transformation. Like PUs, a CU can be subdivided into TUs of different sizes during the residual transformation stage, as illustrated in Figure 3. The selection of TU partitioning within a CU is guided by RDO to achieve an optimal trade-off between coding efficiency and video quality. In particular, for intra prediction, a CU of size 64 × 64 is mandatorily split into four TUs. Likewise, when the PU partition mode is N × N, the corresponding CU is forcibly divided into four TUs during the residual transformation process.

**Figure 3:**
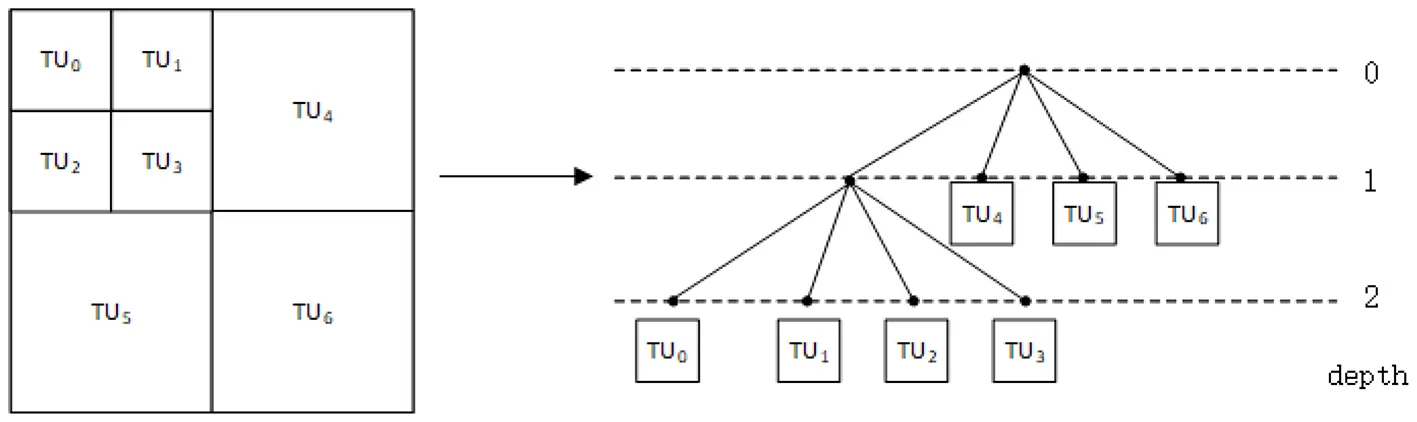
An example of TU partition.

Figure 4 illustrates all partitioning modes of the CU, PU, and TU for the example video frame. In the figure, the partitioning of CUs and PUs is indicated by black lines, while the TUs are delineated using red lines.

**Figure 4:**
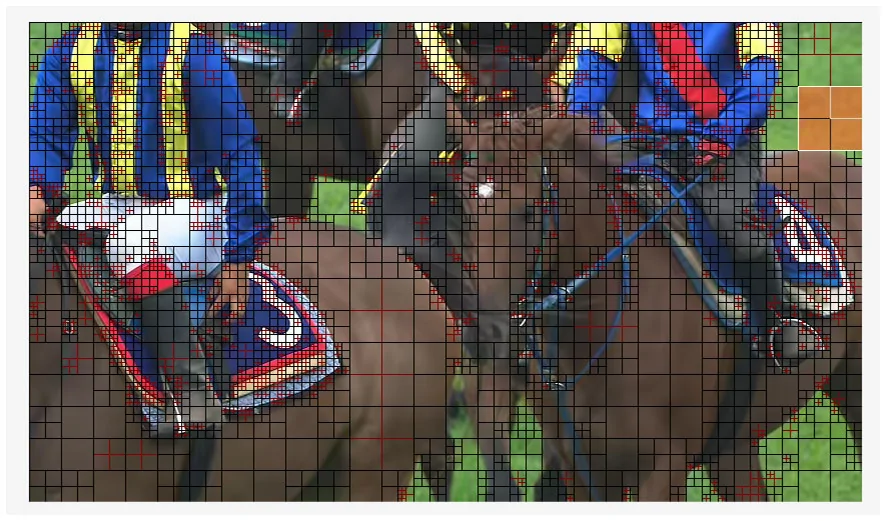
An example of CU PU and TU partition (image adapted with permission from xiph.org, https://media.xiph.org/video/derf/).

### 2.2 Distortion analysis

#### 2.2.1 Distortion of HEVC intra encoding

The intra encoding procedure in the HEVC encoder primarily involves prediction, transformation, quantization, and entropy coding. In this study, our focus is on examining the distortion of the same CU during encoding, taking into account both the stego video and the corresponding cover video. Let *Y* represent the input pixel values of a CU in the cover video. The prediction operation performed by the HEVC encoder for this CU can then be expressed as follows:


$$\begin{array}{l} Y=P_{ori}+R_{ori} \end{array}$$ (4)

where, *P*_*ori*_ denotes the predicted value of the CU, and *R*_*ori*_ represents its residual value.

In the subsequent stages of HEVC intra encoding, the residual *R*_*ori*_ undergoes DCT/DST transformation, followed by quantization and entropy coding. The resulting bitstream for this CU can be represented as *B*_*ori*_, defined by:


$$\begin{array}{lll} B_{ori} & = & Ent\left(\left[\frac{Trans\left(R_{ori}\right)}{Q_{step}}\right]\right) \end{array}$$ (5)

$$\begin{array}{lll} Q_{step} & = & 2^{\frac{QP-4}{6}} \end{array}$$ (6)

In this expression, *Ent*(·) denotes the entropy encoding process, *Trans*(·) represents the DCT or DST transformation, *Q*_*step*_ is the quantization step size, *QP* stands for the quantization parameter, and [, ·, ] indicates the rounding operation. The intra decoding process of HEVC, which is the inverse of the intra encoding procedure, can be formulated as follows:


$$\begin{array}{l} \begin{array}{cc} \bar{Y} & =P_{ori}+{\bar{R}}_{ori}\\ =P_{ori}+\left[Trans^{-1}\left(Q_{step}\times Ent^{-1}\left(B_{ori}\right)\right)\right] \end{array} \end{array}$$ (7)

where, $\bar{Y}$ denotes the reconstructed pixel values of the CU, and ${\bar{R}}_{ori}$ represents the decoded residual of the coding unit. Additionally, *Ent*^−1^(·) indicates the entropy decoding process, while *Trans*^−1^(·) corresponds to the inverse discrete cosine transform transformation.

Ultimately, the distortion produced by the high efficiency video coding encoder can be represented by *ERR*_*ori*_, as defined by the following formula:


$$\begin{array}{l} \begin{array}{cc} ERR_{ori} & =Y-\bar{Y}\\ =P_{ori}+R_{ori}-\left(P_{ori}+{\bar{R}}_{ori}\right)\\ =R_{ori}-{\bar{R}}_{ori}\\ =R_{ori}-\left[Trans^{-1}\left(Q_{step}\times Ent^{-1}\left(B_{ori}\right)\right)\right] \end{array} \end{array}$$ (8)

As indicated by the above equations, HEVC encoder distortion primarily results from rounding and truncation errors.

#### 2.2.2 Distortion of IPM-based steganography

Accordingly, as outlined above, the HEVC encoding procedure for stego video and the distortion introduced by IPM-based steganography can be formulated as follows.


$$\begin{array}{l} Y=P_{ste}+R_{ste} \end{array}$$ (9)

where, *R*_*ste*_ denotes the residual value of the CU after applying IPM-based steganography, *P*_*ste*_ denotes the predict value of the CU after applying IPM-based steganography. In the subsequent intra encoding process, this residual undergoes DCT/DST transformation, followed by quantization and entropy coding. The resulting bitstream for the stego CU can be represented as *B*_*ste*_, defined by:


$$\begin{array}{l} B_{ste}=Ent\left(\left[\frac{Trans\left(R_{ste}\right)}{Q_{step}}\right]\right) \end{array}$$ (10)

Following the intra decoding process in HEVC, which reverses the intra encoding procedure, the reconstructed pixel values of the stego coding unit is expressed as follows:


$$\begin{array}{l} \begin{array}{cc} \bar{Y} & =P_{ste}+{\bar{R}}_{ste}\\ =P_{ste}+\left[Trans^{-1}\left(Q_{step}\times Ent^{-1}\left(B_{ste}\right)\right)\right] \end{array} \end{array}$$ (11)

The distortion induced by IPM-based steganography is quantified by *ERR*_*ste*_, defined as follows.


$$\begin{array}{l} \begin{array}{cc} ERR_{ste} & =Y-\bar{Y}\\ =P_{ste}+R_{ste}-\left(P_{ste}+{\bar{R}}_{ste}\right)\\ =R_{ste}-{\bar{R}}_{ste}\\ =R_{ste}-\left[Trans^{-1}\left(Q_{step}\times Ent^{-1}\left(B_{ste}\right)\right)\right] \end{array} \end{array}$$ (12)

As derived from the preceding equations, distortion in IPM-based steganography primarily arises from rounding and truncation errors. The resulting discrepancy between the stego CU and its corresponding cover CU, denoted △*ERR*, is expressed as follows.


$$\begin{array}{l} \begin{array}{cc} △ERR & =|ERR_{ori}-ERR_{ste}|\\ =||R_{ori}-{\bar{R}}_{ori}|-|R_{ste}-{\bar{R}}_{ste}|| \end{array} \end{array}$$ (13)

Based on the equations of intra encoding in the HEVC encoder and IPM-based steganography, it is observed that although IPM-based steganography embeds secret messages by modifying the intra prediction modes, the distortion caused by steganography is not significant in the spatial domain. Consequently, existing steganalysis algorithms in the spatial domain are unable to identify video steganography based on IPM.

#### 2.2.3 Distortion of block structure-based steganography

When block structure-based steganography is applied to a given CU, its partitioning structure may change, for instance, from no further subdivision to being split into four smaller CUs. Such modifications affect the prediction process of the CU, yielding a new predicted value denoted as *P*_*ste*_. Although block partitioning-based steganography embeds secret information by altering the intra block partitioning, the intra prediction and quantization procedures themselves remain unchanged. Accordingly, the residual value of this CU can be computed using the following formula:


$$\begin{array}{l} Y=P_{ste}+R_{ste} \end{array}$$ (14)

$$\begin{array}{l} P_{ste}=[\begin{array}{cc} P_{spilt1} & P_{spilt2}\\ P_{spilt3} & P_{spilt4} \end{array}]\\ R_{ste}=[\begin{array}{cc} R_{spilt1} & R_{spilt2}\\ R_{spilt3} & R_{spilt4} \end{array}] \end{array}$$

where *R*_*ste*_ denotes the residual corresponding to this coding unit. after the processing of block structure-based steganography. *P*_*spilt*1_, *P*_*spilt*2_, *P*_*spilt*3_, *P*_*spilt*4_ represent the four prediction value of the CUs after further partitioning. *R*_*spilt*1_, *R*_*spilt*2_, *R*_*spilt*3_, *R*_*spilt*4_ denote the four residual values of the coding units following additional partitioning.

In the subsequent intra encoding stage, the residual of this CU undergoes DCT/DST transformation, followed by quantization and entropy coding. The resulting bitstream of the stego CU can be denoted as *B*_*ste*_ and is expressed as:


$$\begin{array}{lll} B_{spilt1} & = & Ent\left(\left[\frac{Trans\left(R_{spilt1}\right)}{Q_{step}}\right]\right) \end{array}$$ (15)

$$\begin{array}{lll} B_{spilt2} & = & Ent\left(\left[\frac{Trans\left(R_{spilt2}\right)}{Q_{step}}\right]\right) \end{array}$$ (16)

$$\begin{array}{lll} B_{spilt3} & = & Ent\left(\left[\frac{Trans\left(R_{spilt3}\right)}{Q_{step}}\right]\right) \end{array}$$ (17)

$$\begin{array}{lll} B_{spilt4} & = & Ent\left(\left[\frac{Trans\left(R_{spilt4}\right)}{Q_{step}}\right]\right) \end{array}$$ (18)

$$\begin{array}{cc} B_{ste}= & B_{spilt1}+B_{spilt2}+B_{spilt3}+B_{spilt4}\\ & Ent[\frac{Trans(R_{spilt1})}{Q_{step}}]+Ent[\frac{Trans(R_{spilt2})}{Q_{step}}] \end{array}\begin{array}{ll} + & Ent[\frac{Trans(R_{spilt3})}{Q_{step}}]+Ent[\frac{Trans(R_{spilt4})}{Q_{step}}] \end{array}$$ (19)

where *B*_*spilt*1_, *B*_*spilt*2_, *B*_*spilt*3_, *B*_*spilt*4_ represent the four bit stream of the CUs after further partitioning.

After HEVC intra decoding, which reverses the intra encoding process, the reconstructed pixel values of the stego CU can be represented as follows:


$$\bar{Y}=\left[\begin{array}{cc} {\bar{Y}}_{spilt1} & {\bar{Y}}_{spilt2}\\ {\bar{Y}}_{spilt3} & {\bar{Y}}_{spilt4} \end{array}\right]$$

$$\begin{array}{l} \begin{array}{cc} {\bar{Y}}_{spilt1} & =P_{spilti}+{\bar{R}}_{spilt1}\\ =P_{spilt1}+\left[Trans^{-1}\left(Q_{step}\times Ent^{-1}\left(B_{spilt1}\right)\right)\right] \end{array} \end{array}$$ (20)

$$\begin{array}{l} \begin{array}{cc} {\bar{Y}}_{spilt2} & =P_{spilti}+{\bar{R}}_{spilt2}\\ =P_{spilt2}+\left[Trans^{-1}\left(Q_{step}\times Ent^{-1}\left(B_{spilt2}\right)\right)\right] \end{array} \end{array}$$ (21)

$$\begin{array}{l} \begin{array}{cc} {\bar{Y}}_{spilt3} & =P_{spilti}+{\bar{R}}_{spilt3}\\ =P_{spilt3}+\left[Trans^{-1}\left(Q_{step}\times Ent^{-1}\left(B_{spilt3}\right)\right)\right] \end{array} \end{array}$$ (22)

$$\begin{array}{l} \begin{array}{cc} {\bar{Y}}_{spilt4} & =P_{spilt4}+{\bar{R}}_{spilt4}\\ =P_{spilt4}+\left[Trans^{-1}\left(Q_{step}\times Ent^{-1}\left(B_{spilt4}\right)\right)\right] \end{array} \end{array}$$ (23)

Ultimately, the distortion introduced by block structure-based steganography can be quantified using *ERRste*, as defined by the following formula.


$$\begin{array}{l} \begin{array}{cc} ERR_{ste} & =Y-\bar{Y}\\ =Y-\left[\begin{array}{c} {\bar{Y}}_{spilt1}{\bar{Y}}_{spilt2}\\ {\bar{Y}}_{spilt3}{\bar{Y}}_{spilt4} \end{array}\right]\\ =P_{ste}+R_{ste}-\left(P_{ste}+{\bar{R}}_{ste}\right)\\ =R_{ste}-{\bar{R}}_{ste}\\ =\left[\begin{array}{c} R_{spilt1}R_{spilt2}\\ R_{spilt3}R_{spilt4} \end{array}\right]-\left[\begin{array}{c} {\bar{R}}_{spilt1}{\bar{R}}_{spilt2}\\ {\bar{R}}_{spilt3}{\bar{R}}_{spilt4} \end{array}\right] \end{array} \end{array}$$ (24)

According to the preceding equations, the distortion caused by block structure-based steganography mainly arises from errors introduced during rounding and truncation. The discrepancy between the stego CU and its corresponding cover CU, denoted as △*ERR*, is given by the following formula:


$$\begin{array}{l} \begin{array}{cc} △ERR & =|ERR_{ori}-ERR_{ste}|\\ =||R_{ori}-{\bar{R}}_{ori}|-|\left[\begin{array}{c} R_{spilt1}R_{spilt2}\\ R_{spilt3}R_{spilt4} \end{array}\right]-\left[\begin{array}{c} {\bar{R}}_{spilt1}{\bar{R}}_{spilt2}\\ {\bar{R}}_{spilt3}{\bar{R}}_{spilt4} \end{array}\right]|| \end{array} \end{array}$$ (25)

Based on the equations of intra encoding in the HEVC encoder and block partitioning-based steganography, it is observed that although block partitioning-based steganography embeds secret messages by modifying the block partitioning, the distortion caused by steganography is not significant in the spatial domain. Consequently, existing steganalysis algorithms in the spatial domain are incapable of detecting block partitioning-based steganography.

Additionally, since block partitioning-based steganography only modified the intra block partitioning structure by manipulating the rate distortion cost, the majority of procedures in intra prediction and quantization coefficients remain unchanged. Therefore, the optimality of intra prediction modes and DCT/DST coefficients is preserved. Hence, existing steganalysis algorithms designed for intra prediction modes and DCT/DST coefficients are ineffective in detecting block partitioning-based steganography.

#### 2.2.4 Distortion of TU-based steganography

When TU-based steganography is applied to a given CU, the TU partitioning may change, for example, from no further subdivision to a division into four smaller TUs. Although this steganographic method embeds secret information by altering the TU partitioning, the intra prediction and quantization processes remain unchanged. Because the intra prediction modes remain unchanged, the predicted values produced by intra prediction are also preserved. Consequently, the residual value of this CU can be computed using the following formula:


$$\begin{array}{l} Y=P_{ori}+R_{ori} \end{array}$$ (26)

During the following intra encoding stage in HEVC, the residual of this CU is transformed via DCT/DST, then quantized and entropy-coded. The resulting bitstream of the stego CU, denoted *B*_*ste*_, can be represented as:


$$\begin{array}{l} \begin{array}{c} B_{ste}=Ent\left[\frac{Trans\left(Res_{spilt1}\right)}{Q_{step}}\right]+Ent\left[\frac{Trans\left(Res_{spilt2}\right)}{Q_{step}}\right]+\\ Ent\left[\frac{Trans\left(Res_{spilt3}\right)}{Q_{step}}\right]+Ent\left[\frac{Trans\left(Res_{spilt4}\right)}{Q_{step}}\right] \end{array} \end{array}$$ (27)

where, *Res*_*split*1_, *Res*_*split*2_, *Res*_*split*3_, and *Res*_*split*4_ represent the residual values of the four TUs after additional partitioning. After HEVC intra decoding, which reverses the intra encoding process, the reconstructed pixel values of the stego CU are given by:


$$\begin{array}{l} \begin{array}{cc} \bar{Y} & =P_{ori}+{\bar{R}}_{ste}\\ =P_{ori}+\left[Trans^{-1}\left(Q_{step}\times Ent^{-1}\left(B_{ste}\right)\right)\right] \end{array} \end{array}$$ (28)

Ultimately, the distortion introduced by TU-based steganography can be quantified using *ERRste*, as defined by the following formula:


$$\begin{array}{l} \begin{array}{cc} ERR_{ste} & =Y-{\bar{Y}}_{ste}\\ =P_{ori}+R_{ori}-\left(P_{ori}+{\bar{R}}_{ste}\right)\\ =R_{ori}-{\bar{R}}_{ste}\\ =R_{ori}-\left[Trans^{-1}\left(Q_{step}\times Ent^{-1}\left(B_{ste}\right)\right)\right] \end{array} \end{array}$$ (29)

According to the preceding equations, the distortion caused by TU-based steganography mainly arises from rounding and truncation errors. The discrepancy between the stego CU and its corresponding cover CU, denoted △*ERR*, is defined by the following formula:


$$\begin{array}{l} △ERR=|ERR_{ste}-ERR_{ori}|\\ =|(R_{ori}-{\bar{R}}_{ste})-(R_{ori}-{\bar{R}}_{ori})|\\ =|{\bar{R}}_{ori}-{\bar{R}}_{ste}|=\\ |Trans^{-1}(Q_{step}\times Ent^{-1}(B_{ori}))-\\ Trans^{-1}(Q_{step}\times Ent^{-1}(B_{ste}))|\\ =|Trans^{-1}(Q_{step}\times Ent^{-1}(Ent([\frac{Trans(R_{ori})}{Q_{step}}])))\\ -Trans^{-1}(Q_{step}\times Ent^{-1}(Ent[\frac{Trans(Res_{spilt1})}{Q_{step}}]+\\ Ent[\frac{Trans(Res_{spilt2})}{Q_{step}}]+Ent[\frac{Trans(Res_{spilt3})}{Q_{step}}]+\\ Ent[\frac{Trans(Res_{spilt4})}{Q_{step}}]))| \end{array}$$ (30)

Based on the equations of intra encoding in the HEVC encoder and TU-based steganography, it is observed that although TU-based steganography embeds secret messages by modifying the TU partition mode, the distortion caused by steganography is not significant in the spatial domain. Consequently, existing steganalysis algorithms in the spatial domain are incapable of detecting TU-based steganography.

Additionally, since TU-based steganography only modified the TU partition modes by manipulating the rate distortion cost, the majority of procedures in intra prediction remain unchanged. Therefore, the optimality of intra prediction modes is preserved. Hence, existing steganalysis algorithms designed for intra prediction modes are ineffective in detecting TU-based steganography.

## 3 The IPM map

Based on the analysis in the previous section, we can conclude that these three different kinds of intra frame steganography algorithms will not have a significant impact on the spatial domain of the video. So, current steganalysis algorithms mainly determine whether a video has undergone steganography by detecting the encoding information of different carriers, such as IPM, partitioning block structure. Therefore, the majority of steganalysis algorithms can detect and only can detect one specific types of video steganalysis algorithms.

### 3.1 The IPM map

To simultaneously detect these different types of video steganography algorithms embedded in various carrier types, it is necessary to extract information that can jointly characterize the optimality of both the partitioning structure of the carrier video and the intra prediction modes for subsequent steganalysis. Inspired by the concept in Dai et al. (2023), this paper proposes IPM map. As shown in Figure 5A, this is an example of a 32 × 32 CU that is partitioned into four 16 × 16 CUs, with the upper right 16 × 16 CU further partitioned into four 8 × 8 CUs. The uppermost left 8 × 8 CU adopts the NxN PU partitioning mode, and the values indicated on these CUs represent the IPM values of each CU. Since 4 × 4 is the minimum coding unit in HEVC, each video frame is divided into multiple non-overlapping 4 × 4 units, where the value of each unit corresponds to the IPM value of the PU it belongs to. As illustrated in Figure 5B, this is the IPM map (of size 8 × 8) corresponding to the example CU. It can be observed that the IPM structure map not only represents all the IPM information within the CU but also reflects the CU's partitioning structure. This is because larger CUs will present identical values over a larger region in the corresponding position of the IPM map, whereas smaller CUs are more likely to exhibit varying values in the surrounding positions of the IPM map. Following this approach, an IPM structure map for an entire video frame can be obtained and subsequently used for steganalysis.

**Figure 5:**
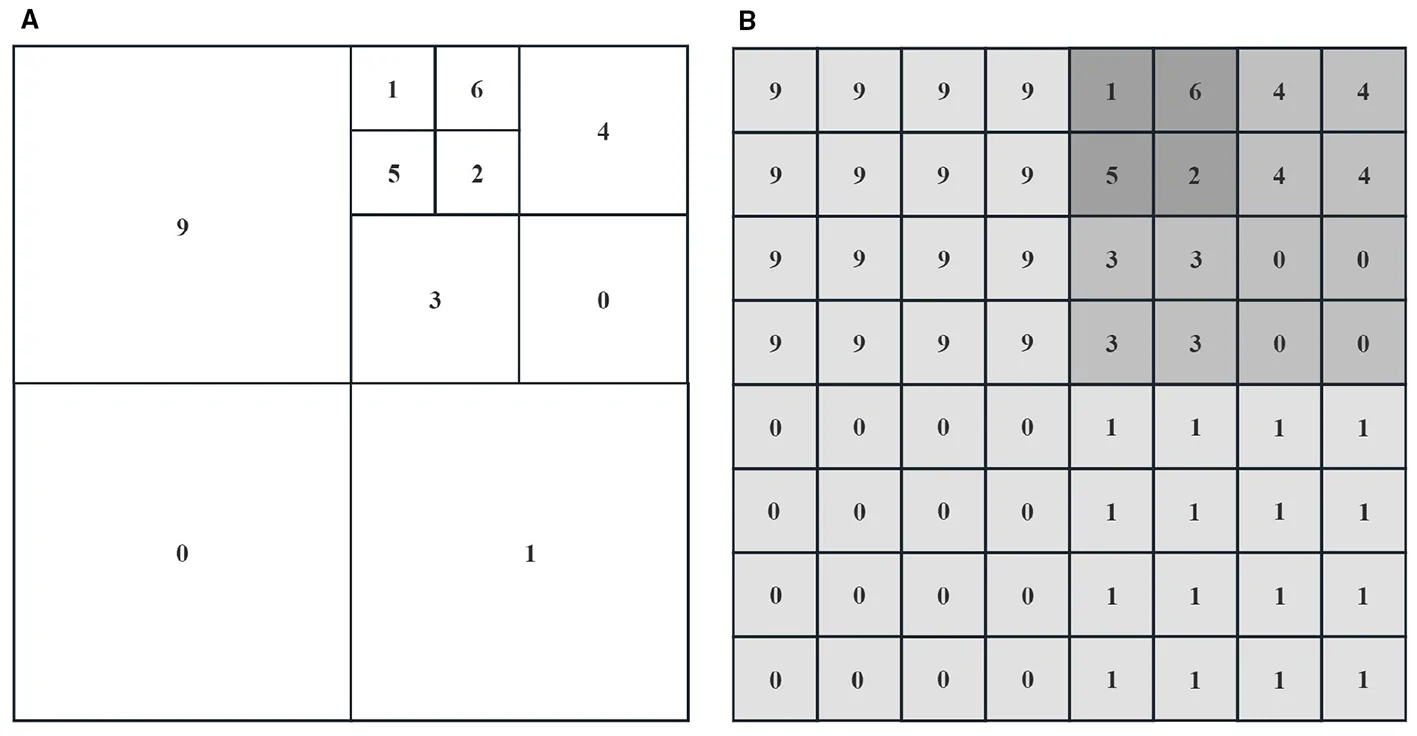
**(A)** The example CU. **(B)** IPM map of the example CU.

### 3.2 Embedding effect on IPM map

Based on the analysis in the previous chapter, it can be observed that none of these three types of intra-based video steganography algorithms have a significant impact on the spatial domain of the video. Steganography methods based on partition structures and TU modes disrupt the optimality of the partitioning structure in certain regions of the cover video, while steganography algorithms based on intra-prediction modes compromise the optimality of the IPM.

Algorithm 2 Generation of joint histogram 
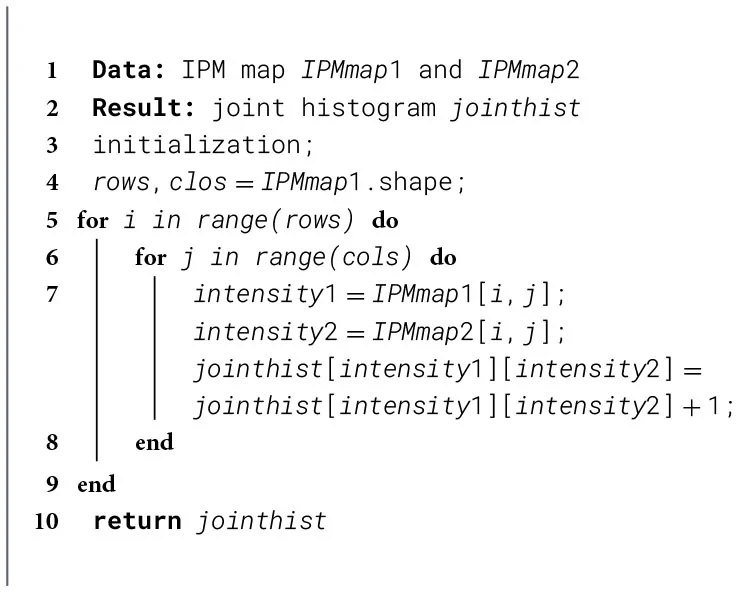

To verify the above viewpoint, we analyzed numerous videos and selected “basketball.YUV” as an example video to examine the impact of intra-frame block structure-based video steganography algorithms on the block structure of the video. The steganography algorithm employed in the experiment was Dong et al. (2023b), with QP set to 8, and the experimental environment consisted of X265 and HM. We calibrated the original video and extracted the IPM maps of both the original video and the calibrated video. Following Algorithm 2 and Formula 31 we computed the joint histogram of these two IPM maps and then calculated their probability transition matrix according to the corresponding formula. Similarly, we calibrated the stego video and extracted the IPM structure maps of both the stego video and its calibrated video, and calculated the probability transition matrix for these two IPM maps.


$$\begin{array}{l} Matrix\left[i\right]\left[j\right]=\frac{jointhist\left[i\right]\left[j\right]}{\sum_{j=0}^{34}jointhist\left[i\right]\left[j\right]} \end{array}$$ (31)

As shown in Figures 6A, B, we visualized the two probability transition matrices, where brighter colors indicate values closer to 1 and darker colors represent values closer to 0. It can be observed that in both matrices, the diagonal values are very close to 1, indicating that regardless of whether steganography has been applied, the majority of IPMs remain unchanged after calibration. The off-diagonal values are mainly concentrated in the second column, demonstrating that whether or not steganography is applied, some intra-prediction mode values tend to be 1 after calibration. This is because the final intra-prediction mode is typically determined through two rounds of recursive selection: an initial coarse screening that rapidly generates a list of possible intra-prediction modes, followed by a final selection. Prediction mode 1 will always appear in the possible prediction mode list for final iteration, regardless of its cost ranking in the coarse screening. Additionally, when the intra-prediction mode value is 1, it corresponds to the DC prediction mode, which is generally suitable for smooth regions of the image. Whether or not steganography is applied, the video inevitably loses some fine details after the first encoding, which can lead to certain regions appearing smoother compared to the original YUV video. As a result, the intra-prediction mode may shift to 1 after calibration. Finally, aside from the diagonal and the second column, most other regions in the probability transition matrix have very low values; however, in these regions, the values for the stego video are noticeably higher than those for the non-stego video. This indicates that compared to the cover video, stego video disrupts the trend of IPM probability transitions after calibration. Therefore, by calibrating the video under examination and analyzing changes in its IPM map, it is possible to determine whether the video has been subjected to steganography.

**Figure 6:**
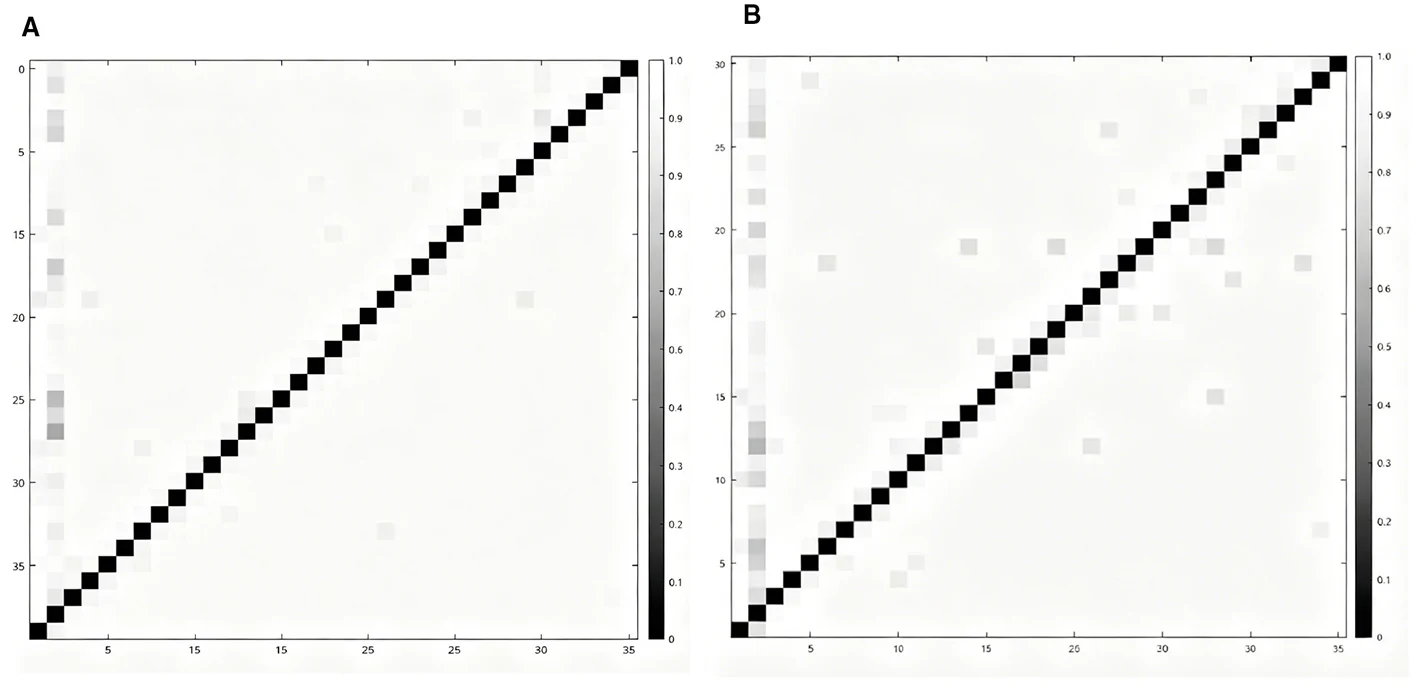
**(A)** Probability transition matrix of cover video. **(B)** Probability transition matrix of stego video.

## 4 The proposed method

In this section, we introduce the architecture of IFSN.

### 4.1 The overall architecture

Drawing on the analysis presented in the previous section, it can be concluded that determining whether a video has undergone intra-frame steganography by analyzing the differences between the IPM maps of the original video and its calibrated video is feasible. After calibrating the video, the IPM maps of both the original video and its calibrated video are extracted. The difference between these two IPM maps is then computed to generate a difference map (DI map), which is used as input to a deep residual network for steganalysis.


DIMapi=Abs(IPMmapi−IPMmapi') (32)

where *IPMmap*_*i*_ represents the IPM map of original video, $IPMmap_{i}\text{'}$ represents the IPM map of the calibrated video.

The network architecture designed in this study is primarily based on a fine-tuned ResNet18 structure, with the integration of the ECA attention mechanism. This is because convolutional residual networks ultimately perform a global average pooling operation, which means that each channel's final resolution is only 1 × 1. In other words, spatial differences within each channel are not critical, and the final result mainly depends on the contributions of all channels; therefore, the weights assigned to different channels are highly significant. The core idea of ECA is to introduce a channel attention mechanism into the convolution operation to enhance the representational capacity of the features. The channel attention mechanism aims to adaptively adjust the weights of the channel features, enabling the network to better focus on important features while suppressing less relevant ones. Through this mechanism, ECA effectively strengthens the network's representational capability while avoiding excessive increases in parameters and computational costs. In summary, based on the ResNet18 architecture, this study fine-tunes the network and incorporates the ECA attention mechanism to serve as the steganalysis network.

As shown in Figure 7, the entire network architecture consists of three parts: feature extraction, feature representation, and binary classification. The feature extraction component is composed of preprocessing and BasicBlocks. The feature representation component consists of two PoolingBlocks and a global average pooling layer. The binary classification component is made up of a fully connected layer and a softmax layer.

**Figure 7:**
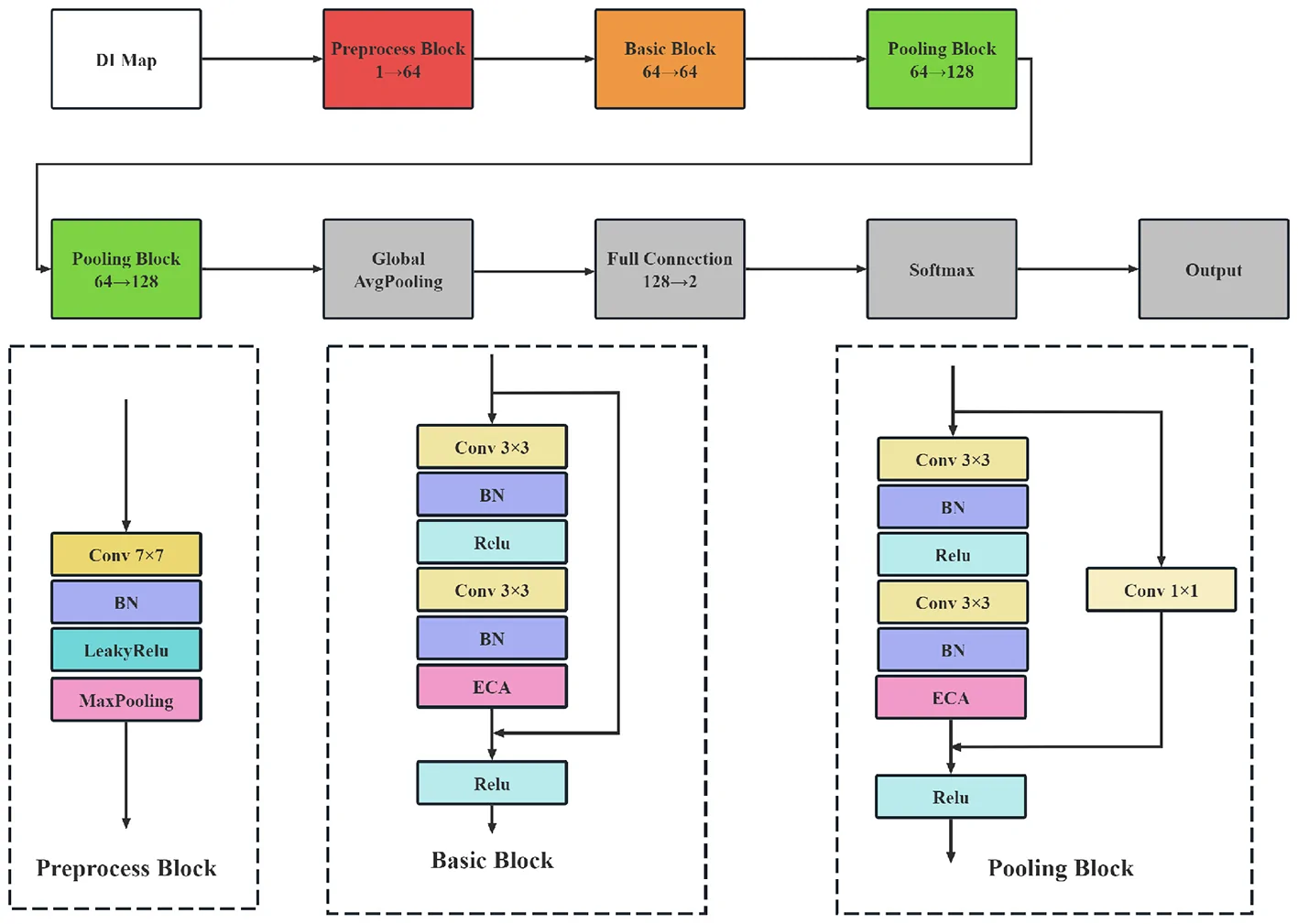
The overall framework of the IFSN.

### 4.2 Preprocessing

Firstly, a preprocessing layer is employed to extract steganographic noise, thereby facilitating subsequent processing. The preprocessing layer consists of a 7 × 7 convolutional layer (stride=2, padding=3), batch normalization (BN), a leaky ReLU activation function, and a 3 × 3 max pooling layer (stride=2, padding=1). The ReLU activation function is widely used in convolutional neural networks; however, it truncates all values below zero. To make the steganographic noise more explicitly represented, the preprocessing layer in this study uses the leaky ReLU activation function instead.

In general, deep learning-based image steganalysis algorithms often utilize the Spatial Rich Model (SRM) as the convolutional kernels in the preprocessing layer. However, this approach may not be suitable for the algorithm proposed in this study. This is because most deep learning-based image steganalysis algorithms take the image to be analyzed directly as the input to the steganalysis network, and the high-pass filtering kernels in SRM can effectively extract steganographic noise from the image. In contrast, the proposed method does not feed the raw video frames directly into the convolutional residual network but instead uses the DI map of the video to be analyzed as the input. This is because video steganography algorithms based on IPM, TU modes, or block structures typically have minimal impact on the spatial domain of the cover video. As such, directly using the pixel values of the video frames as input to the residual network may not effectively detect whether the video has been subjected to steganography.

### 4.3 Feature extraction

Since performing pooling operations in the early layers of a convolutional network may lead to the loss of local information in feature extraction, which can be detrimental to the learning of the entire network. Many deep learning-based steganalysis algorithms for images or videos typically use three Basic Blocks to extract steganographic noise features. Since the DI map used as input in this study has already undergone a certain degree of feature extraction, only one Basic Block is used for feature extraction. As shown in Figure 7, the Basic Block consists of a 3 × 3 convolutional layer (stride=1, padding=1), BN, ReLU, another 3 × 3 convolutional layer (stride=1, padding=1), BN, and an ECA attention module. Moreover, the use of shortcut connections allows the network to capture residual relationships between inputs and outputs, thereby improving the optimization of deeper layers.

### 4.4 Feature representation

The objective of the feature representation layer is to reduce the size of the feature maps and the number of network parameters while enhancing the network's representational capacity. As shown in the Figure 7, the feature representation layer consists of two PoolingBlocks and a Global Average Pooling module. Each PoolingBlock comprises a 3 × 3 convolutional layer (stride=2, padding=1), batch normalization (BN), ReLU, a 3 × 3 convolutional layer (stride=1, padding=1), BN, and an ECA attention module. To maintain the shortcut connections of the residual structure, a 1 × 1 convolutional layer is employed to ensure consistency between the dimensions of the input and the feature maps after the ECA attention module. Each time a feature map passes through a PoolingBlock, its spatial dimensions are halved while the number of channels is doubled. After passing through the two PoolingBlocks, the feature map undergoes global average pooling and is transformed into a 128-dimensional feature vector.

### 4.5 Binary classification

In the binary classification layer, we perform binary classification on the feature vector using a fully connected layer and a Softmax function to obtain the final output result.

## 5 Experimental results

### 5.1 Experimental setting

This experiment was conducted using 28 uncompressed video sequences, comprising approximately 14,000 frames in total. The resolution of the video sequence is 1,920 × 1,080. The HM16.15 reference software was employed, with a GOP structure configured as IPPP. The dataset was divided into training and test sets at an 80:20 ratio, ensuring that frames from the same YUV video were assigned exclusively to either the training or test set to avoid potential bias from repeated content. The experimental results were not obtained under the cross-dataset validation. The model was trained with a learning rate of 0.0001 and a batch size of 32 over 50 epochs, with the learning rate decayed by a factor of 0.5 every 10 epochs.

The detection performance can be evaluated using the expression in the following formula:


$$\begin{array}{l} P_{Acc}=\frac{TP+TN}{T+F} \end{array}$$ (33)

where *P*_*Acc*_ denotes the final detection accuracy rate; *TP* denotes the count of stego videos correctly identified, *TN* represents the number of correctly predicted cover-videos. *T* represents the number of the stego-videos, *F* represents the number of the cover-videos.

For most video steganography algorithms, the payload is calculated using the following formula:


$$\begin{array}{l} Payload=\frac{Num_{Mes}}{Num_{Cover}} \end{array}$$ (34)

where *Num*_*Mes*_ denotes the number of embedded secret messages, *Num*_*Cover*_ denotes the number of covers.

For intra block partitioning-based Table 1, Tew's algorithm embeds secret bits through alterations in the block partitioning structure of 8 × 8 CUs (Tew and Wong, 2014). In Dong's method (Dong et al., 2023a), the secret messages are embedded into multi kinds of CU with the structure merging strategy. Yang's method embeds the secret messages by using quad-tree structure-preserving adaptive steganography (Yang et al., 2024). In addition, some intra block partitioning-based steganography methods, such as Dong's and Tew's video steganography are not designed for adjustable payload. In Tew's method, all of the 8 × 8 CU are selected as covers and one secret message is embedded in each cover. The payload of Tew's method can be considered as 1. In Dong's method, the secret messages are embedded into multi kinds of CU with the structure merging strategy. Payload in this method is flexible during different videos or different messages. So, payload is not used in our experiments for Dong's method.

**Table 1:** Steganography methods.

Author	Title	Target domain
Tew	Information hiding in hevc standard using adaptive coding block size decision	Block partitioning
Dong	Adaptive hevc steganography based on steganographic compression efficiency degradation model	Block partitioning
Yang	Quad-tree structure-preserving adaptive steganography for hevc	Block partitioning
Xing	A multi-embedding domain video steganography algorithm based on tu partitioning and intra prediction mode	IPM and TU
Dong	Multi-channel hevc steganography by minimizing ipm steganographic distortions	IPM
Saberi	An efficient data hiding method using the intra prediction modes in hevc	IPM
Zhao	A video steganography method based on transform block decision for H.265/HEVC	TU

For IPM-based Table 2, Dong's method (Dong et al., 2023b) modified the IPMs by using an adaptive cost function in multi channels. (Xing et al. 2024) proposed an adaptive steganography based on TU partition and IPM. Saberi's method Saberi et al., (2020) is also used for comparison.

**Table 2:** Steganalysis methods.

Author	Title	Target Domain
Zhai	Universal detection of video steganography in multiple domains based on the consistency of motion vectors	PU mode and MV
Dai	HEVC video steganalysis based on PU Maps and multi-scale convolutional residual etwork	PU mode
Sheng	A prediction mode steganalysis detection algorithm for hevc	IPM
Zhao	Video steganalysis based on intra prediction mode calibration	IPM

For TU-based steganography algorithms, Zhao et al. (2021) propose the first TU-based steganography. Xing et al. (2024) proposed an adaptive steganography based on TU partition and IPM. Payload is not used in Zhao's method, threshold T is used for adjust the number of secret bits for each CTU.

To the best of our knowledge, no steganalysis algorithms have been specifically developed for intra block partitioning-based or TU-based steganography. To evaluate the performance of the proposed steganalysis method, we followed the experimental setup described in Yang et al. (2024) and employed video steganography and steganalysis algorithms that were fully consistent with those in the reference study.

For steganalysis methods against IPM-based steganography (Zhao et al., 2016; Qi et al., 2017), Zhao's algorithm (Zhao et al., 2016) leverages changes in intra prediction mode as the proposed feature sets, whereas Sheng's algorithm Qi et al. (2017) employs changes in the number of blocks as the feature sets. Originally designed for H.264/AVC, Zhao's algorithm accommodates the 35 intra mode directions in HEVC (compared to the 9 in H.264). We expand the 13-dimensional (9 + 4) features to 39-dimensional (35 + 4) features, enabling the transplanted algorithm to effectively detect HEVC intra-frame steganography.

For steganalysis algorithms (Zhai et al., 2020), the theoretical foundation involves focusing on modeling the distribution of motion vectors or block partitioning that deviates from what is generated by the encoder. This algorithm aim to capture the additional distributional distortion introduced by steganography.

For steganalysis algorithms (Dai et al., 2023), a video steganalysis method based on PU maps and multi-scale convolutional residual network is proposed. Finally, the detection result is obtained by the voting mechanism.

### 5.2 Steganalytic performance for different methods

Table 3 presents the detection accuracy of different steganalysis algorithms against various steganalysis methods. For steganography algorithms based on block partitioning, the proposed steganalysis algorithm achieves very high detection accuracy. Except for Sheng's method, which can effectively detect Tew's steganography, most existing steganalysis algorithms struggle to detect these two latest block partitioning-based steganography methods. This is primarily because these algorithms were not designed for block partitioning-based steganography. Sheng's algorithm, however, leverages changes in PU structures before and after calibration, and since Tew's method is relatively outdated with a high embedding rate, it can be efficiently detected. For IPM-based steganography, the proposed algorithm also significantly outperforms other approaches in terms of detection accuracy. The methods by Zhai and Dai were not designed for intra-frame IPM steganography, hence they yield limited performance. In the context of TU-based steganography, the proposed steganalysis algorithm again demonstrates superior detection accuracy compared to others, as most existing methods were not tailored to target TU-based steganography. Although Zhao's steganalysis algorithm can effectively detect Xing's method, this is due to the fact that Xing's steganography alters not only TU partitioning modes but also some IPMs, enabling Zhao's method to detect it via IPM-related features. Among all the evaluated steganalysis methods, the proposed approach achieves consistently high detection accuracy across all three types of steganography.

**Table 3:** Steganalytic performance for different methods.

Steganalysis	Block partitioning			IPM			TU	
	Tew	Dong	Yang	Xing	Dong	Saberi	Zhao	Xing
	**Payload** = **1**		**Payload** = **0.5**	**Payload** = **0.5**	**Payload** = **0.5**	**Payload** = **0.5**	**T** = **8**	**Payload** = **0.5**
Zhai	48.13%	51.74%	50.47%	48.82%	49.65%	51.68%	49.87%	48.82%
Dai	50.99%	49.33%	51.31%	48.57%	52.00%	49.68%	48.90%	48.57%
Sheng	97.17%	68.64%	61.77%	77.31%	57.37%	67.45%	61.03%	77.31%
Zhao	70.92%	67.17%	52.99%	83.21%	72.39%	69.12%	59.12%	83.21%
Proposed	98.93%	98.91%	99.42%	99.80%	88.65%	83.37%	99.23%	99.80%

### 5.3 Steganalytic performance for different payloads

According to the results in Table 3, Zhai's and Dai's algorithms perform poorly in detecting block partitioning-based steganography methods. Table 4 further compares steganalytic performance with Zhao's, Sheng's, and the proposed algorithms at different embedding payloads. As discussed earlier, Tew's method effectively has an embedding rate of 1, while Dong's method lacks a defined embedding rate. The results in Table 4 show that, apart from Sheng's method effectively detecting Tew's algorithm, the proposed steganalysis method performs robustly across different block-based steganography techniques. This is because the extracted IPM map in our approach captures not only IPM information but also the video's block partitioning information. Coupled with the IFSN architecture, the proposed method can efficiently detect block partitioning-based steganography.

**Table 4:** Detection accuracy against block partition-based steganography.

**Steganography**	Payload	Sheng	Zhao	Proposed
Yang et al. (2024)	0.1	61.59%	50.98%	98.97%
	0.3	61.73%	51.91%	98.97%
	0.5	61.77%	52.99%	99.42%
Dong et al. (2023a)	/	68.64%	67.17%	98.91%
Tew and Wong (2014)	1	97.17%	70.92%	98.93%

Similarly, Table 5 further investigates the performance of Zhao's, Sheng's, and the proposed algorithm under different embedding rates for IPM-based steganography. The results reveal that Sheng's algorithm performs only moderately in this context. Zhao's method achieves some level of detection but is insufficient, especially at lower embedding rates. In contrast, the proposed algorithm achieves effective detection across all evaluated IPM-based steganography methods.

**Table 5:** Detection accuracy against IPM-based steganography.

Steganography	Payload	Sheng	Zhao	Proposed
Xing et al. (2024)	0.1	65.57%	65.26%	86.03%
	0.3	70.94%	72.35%	99.80%
	0.5	77.31%	83.21%	99.80%
Dong et al. (2023b)	0.1	54.04%	56.12%	83.88%
	0.3	54.52%	67.92%	84.27%
	0.5	57.37%	72.39%	88.65%
Saberi et al. (2020)	0.1	59.01%	55.88%	77.81%
	0.3	65.54%	61.76%	80.05%
	0.5	67.45%	69.12%	83.37%

### 5.4 Steganalytic performance for different QPs

Table 6 further analyzes the performance of the proposed method under different QP settings for detecting various steganographic methods. The results show that regardless of the steganography type, block partitioning-based steganography, IPM-based steganography, or TU-based steganography, the proposed method consistently achieves effective detection. While detection accuracy decreases slightly at higher QP levels, this can be attributed to the encoder's preference for larger coding blocks at higher QP. Since the evaluated steganography methods tend to embed data in smaller blocks, higher QP result in reduced number of embedded message, which slightly reduces the effectiveness of the proposed detection method.

**Table 6:** Detection accuracy for different QPs.

Steganography	Payload	QP = 8	QP = 16	QP = 24
Yang et al. (2024)	0.1	93.60%	79.98%	98.97%
	0.3	98.84%	92.26%	98.97%
	0.5	99.36%	93.28%	99.42%
Dong et al. (2023b)	0.1	93.45%	82.28%	83.88%
	0.3	96.22%	83.95%	84.27%
	0.5	95.33%	88.36%	88.65%
Tew and Wong (2014)	1	99.56%	99.03%	98.93%
Zhao et al. (2021)	T=2	95.69%	93.13%	92.5%
	T=4	98.35%	97.64%	97.98%
	T=8	99.74%	91.36%	99.23%
Xing et al. (2024)	0.1	92.74%	98.71%	86.03%
	0.3	100%	92.00%	99.80%
	0.5	99.80%	91.68%	99.80%
Dong et al. (2023a)	/	97.05%	97.57%	98.91%
Saberi et al. (2020)	0.1	99.74%	94.30%	77.81%
	0.3	99.80%	94.11%	80.05%
	0.5	99.68%	94.69%	83.37%

## 6 Conclusion

Most of the existing video steganalysis algorithms can only detect a single type of video steganography and are unable to detect other types of video steganography. In this paper, a universal video steganalysis algorithm based on convolutional residual network is proposed. After analyzing three different types of video steganography, we found that compared to the cover video, stego video disrupts the trend of IPM probability transitions after calibration. In order to represent both IPM information and block partition information, IPM map is proposed in this paper. The difference between the IPM map of original video and its calibrated video is the input of convolutional residual network IFSN. IFSN is primarily based on a fine-tuned ResNet18 structure, with the integration of the ECA attention mechanism. IFSN consists of three parts: feature extraction, feature representation, and binary classification. Experimental results demonstrate that IFSN effectively detects different video steganography algorithms in multiple domains of HEVC intra frame. Although H.266 introduces more intra prediction modes and more flexible block partitioning structures than H.265, we believe that the ideas proposed in this paper can provide valuable insights for future research. In future work, we will further investigate steganalysis methods for H.266 video encoder and attempt to develop a true universal steganalysis framework capable of detecting all types of H.266 video steganography.

## Data Availability

Publicly available datasets were analyzed in this study. This data can be found here: https://media.xigh.org/video/derf/.
